# Robotic Sigmoidectomy of a Rare Instance of Sigmoid Colon Duplication in an Elderly Patient: A Case Report

**DOI:** 10.7759/cureus.44208

**Published:** 2023-08-27

**Authors:** Gavin H Ward, Alexis Sireci, William Wilder, Alain Soto, Roynny Sanchez, Aruna Dash, Eliyahu Shemesh

**Affiliations:** 1 School of Medicine, St. George's University, True Blue, GRD; 2 General Surgery, Delray Medical Center, Delray, USA; 3 General Surgery, Larkin Community Hospital, Miami, USA; 4 Pathology, Delray Medical Center, Delray, USA

**Keywords:** bowel obstruction, alimentary tract duplication, sigmoidectomy, enteric duplication, robotic surgical procedures

## Abstract

Duplication of the alimentary tract is a rare malformation that can occur in any portion of the gastrointestinal tract. Rarely diagnosed in adulthood, it is usually an incidental finding due to non-specific gastrointestinal symptoms. Approximately 80% of cases are diagnosed in infants less than two years old. The most common location is the ileum, and the least common location is the rectum. Embryological theories discussing the etiology of alimentary tract duplications include failure of internal vacuolization during the sixth week of fetal development and/or the yolk-sac dorsal protrusion may adhere to the ectoderm during somite development. Environmental factors such as trauma or hypoxia affecting various intestinal fragments receiving blood supply can evolve into duplication. Excision with consideration to the common blood supply to protect the native bowel is the preferred treatment approach. We present the case of an elderly 70-year-old male with a bowel obstruction complicated by sigmoid duplication. After preoperative assessments, the patient underwent a robotic sigmoidectomy. This case report highlights colonic duplication as a differential diagnosis in the bowel obstruction of an elderly patient.

## Introduction

Duplication of the alimentary tract is a rare malformation that can occur at any point from the oropharynx to the anal canal. The most common location is the ileum, accounting for 30% of cases, and the least common location is the rectum, accounting for 5 to 5.5% [1,2]. Alimentary tract duplication is most commonly diagnosed in infants less than two years of age and makes up approximately 80% of cases [3]. It is usually an incidental finding in adulthood and is rarely diagnosed. Common signs and symptoms of colonic duplication in all ages include non-specific constipation and abdominal pain. More commonly, adult patients are asymptomatic. Imaging modalities are low in sensitivity in diagnosing alimentary tract duplications, and therefore, diagnoses may not occur until a complication arises. The preferred treatment approach is surgical excision of the duplication with consideration of the common blood supply to the native structure [3,4]. We present the case of an elderly 70-year-old male with a bowel obstruction complicated by sigmoid colon duplication. The duplication was excised via robotic sigmoidectomy.

## Case presentation

A 70-year-old male presented to the emergency department with constant lower abdominal pain, rectal pain, and constipation for four days. He had a past medical history of irritable bowel syndrome, multiple intrahepatic hemangiomas, masses in his spleen, and hypothyroidism, and a patient stated a surgical history of polyp removal during a colonoscopy, umbilical herniorrhaphy, and inguinal hernia repair. It was not specified by the patient if duplication was noted during the prior colonoscopy and polyp removal. The patient has a family history of Hodgkin’s lymphoma and no history of bowel diseases. The patient was a former smoker. On physical examination, the patient was positive for generalized tenderness to palpation of the lower abdomen. The rest of his exam and vitals were unremarkable. Labs on admission showed elevated white blood cell count (12.5), elevated neutrophil percentage (81.7%), increased absolute neutrophils (10.2), and elevated bilirubin (1.6). 

**Figure 1:**
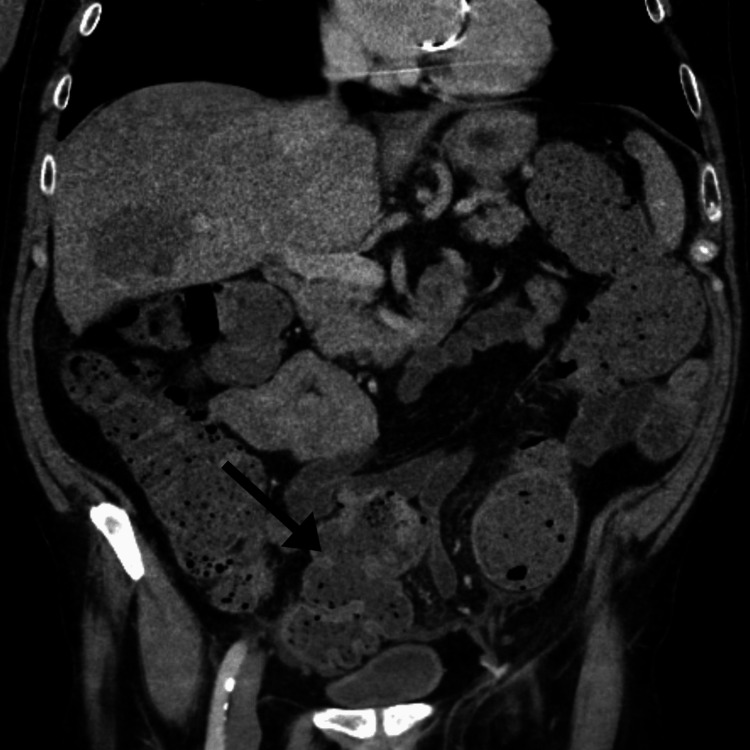
Computed tomography scan in coronal view demonstrating moderate dilation of the sigmoid colon, surrounding inflammatory changes, and luminal wall thickening.

An abdominal CT revealed moderate dilatation of the sigmoid colon and descending colon. A transition point was seen at the level of the distal sigmoid colon. The findings were suggestive of an underlying mass versus stricture. There were surrounding inflammatory changes consistent with impaction (Figure 1).

**Figure 2:**
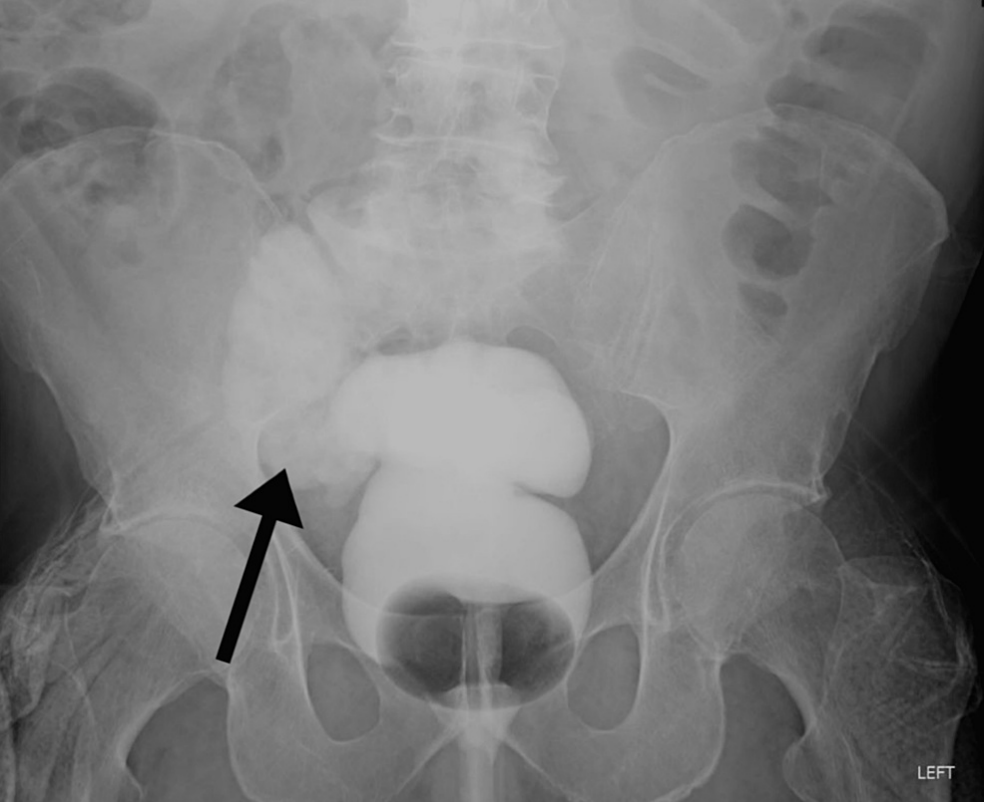
Gastrografin enema demonstrating only a small amount of contrast progressing into the proximal sigmoid colon that is compatible with a stricture at the distal sigmoid colon or rectosigmoid junction.

The abdominal CT was not able to discern the sigmoid duplication. A gastrografin enema was performed due to continued, significant abdominal pain and a stricture of the distal sigmoid or rectosigmoid junction of indeterminate etiology (Figure 2). 

**Figure 3:**
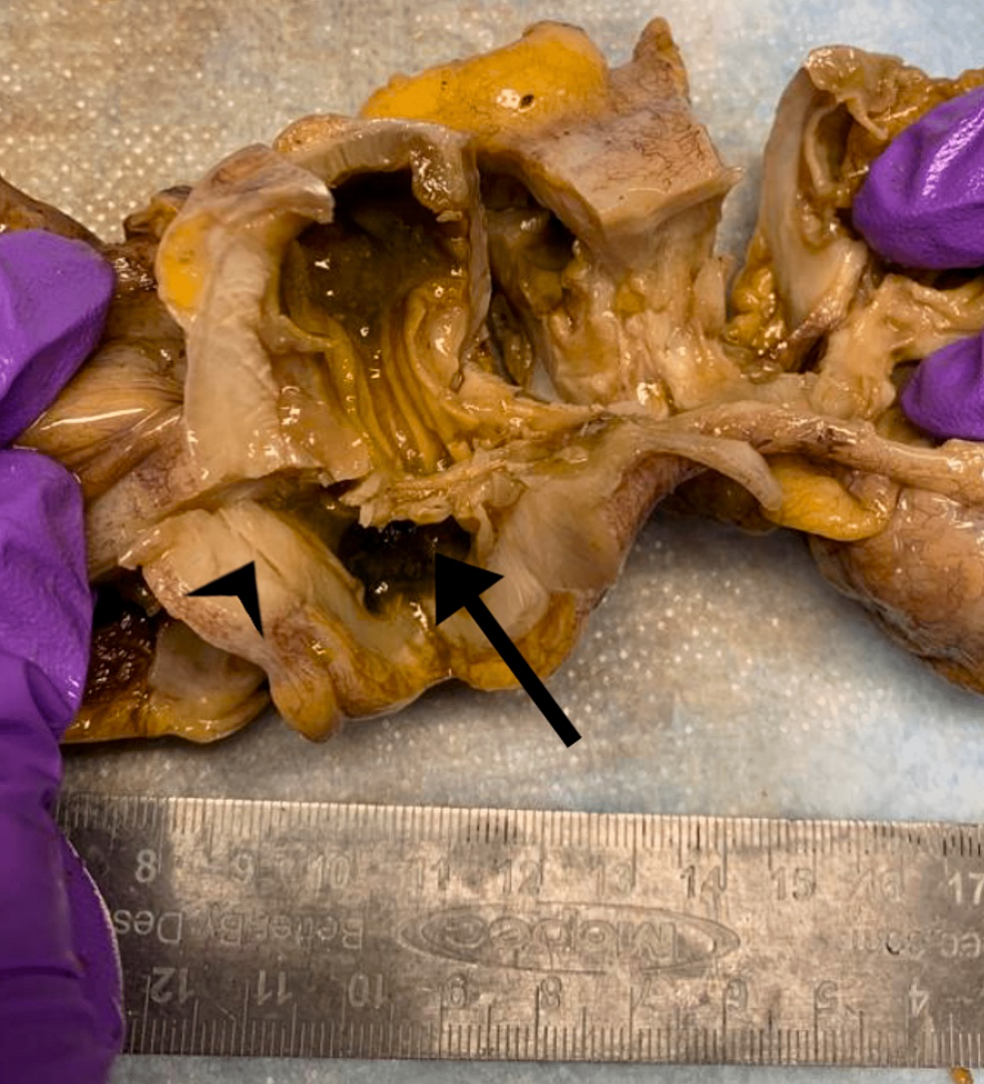
Gross pathology of an excised sigmoid colon demonstrating the sigmoid wall duplication (arrowhead) and its lumen (arrow).

A colonoscopy and sigmoidoscopy were performed for direct visualization. The colonoscopy demonstrated severe diverticular disease of the left colon causing functional obstruction and strictures. A colonic stent was placed to treat the bowel obstruction utilizing fluoroscopic guidance. The colonoscopy was performed and the stent was placed the same day. This obstruction prompted a recommendation for an elective resection of the sigmoid colon. A low anterior robotic sigmoidectomy with primary anastomosis was performed three days after the colonoscopy and stent placement. Intraoperatively, two complete lumens were noticed, indicative of a sigmoid duplication (Figure 3).

His post-operative recovery was uneventful, and the patient was to follow up as an outpatient. Examination of the colon histopathology showed benign colonic mucosa with reduplication of the colon segment and diverticulosis. The margins of the surgical resection were benign colonic mucosa and a viable bowel wall. There was one benign lymph node and no malignancy present. The confirmed diagnosis via pathology was sigmoid colon duplication.

## Discussion

Alimentary duplication is rare and occurs in only one of 4500 live births at a 2:1 ratio of female to male [5]. Alimentary duplication occurs in infants less than two years of age 80% of the time [6-8]. There are only a few cases reporting young adult and adult alimentary duplications. We present the case of an elderly 70-year-old male patient with a colonic duplication. Alimentary duplication can occur anywhere along the digestive tract but occurs most commonly in the order of the ileum (30%), ileocecal valve (30%), duodenum (9.6%), stomach (8.2%), jejunum (8-8.2%), colon (6-7%), and rectum (5-5.5%) [1,6]. Alimentary duplication must satisfy three criteria: (1) a layer of typical or heterotopic alimentary mucosa or cystic forms exists; (2) a smooth muscular layer surrounds it; (3) the walls of the native alimentary tract are continuous with those of its duplication. The duplication’s structure may be tubular, double-barreled, cystic, or spherical, and it shares the same blood supply as the native alimentary tract [6].

The embryological origin of alimentary duplication is the gut tube that develops from the yolk sac’s endodermal lining and therefore the mucosa and submucosa. This is engulfed by the mesoderm and forms the mesenteric vessels, adventitia, lamina propria, and muscularis mucosa. Neural crest cells migrate to the submucosa and become the enteric nervous system. The gut develops into three sections: foregut, midgut, and hindgut. Each section's blood supply is from the celiac artery, superior mesenteric artery, and inferior mesenteric artery and their branches, respectively [9]. Concomitant anomalies associated with alimentary tract duplication include hemivertebra (15% of cases) that may cause spinal cord compression, double bladder, genitourinary, and meningomyelocele [10]. There are several theories discussing the cause of alimentary duplication. Embryologic factors include failure of internal vacuolization forming a single lumen after the lumen obliterates during the sixth week of development or the yolk-sac dorsal protrusion may adhere to the ectoderm during somite development and develop into duplications [6]. There are atypical or ectopic potentials for Meckel's diverticula that are commonly duplications [11]. The most common ectopic potential is gastric mucosa in the walls of the duplication [12]. Environmental factors include trauma and hypoxia, which can cause ischemic necrosis of the intestine and various intestinal fragments receiving blood supply can evolve into duplication [8]. 

The few case reports of adults with gastrointestinal tract duplication range in age from 20 years old to 52 years old [2,5,6,8,13]. Duplications reported in these case reports were of the ascending colon twice, the transverse colon three times, and the descending colon once. Trauma was reported as a history and potential cause of duplication because it cannot be ruled out in the 52-year-old case reported by Peng et al. (2023) [8].

The case of a 70-year-old male with a sigmoid colon duplication highlights the possible relevance of alimentary tract duplication in the elderly and the need for surgical removal due to bowel obstruction. The patient initially presented with abdominal pain, constipation, and rectal pain with a history of a colonic mass. Preoperative assessments of the patient deemed him a good candidate for robotic sigmoidectomy. In order to avoid complications, excision with consideration to the common blood supply to protect the native bowel is the preferred treatment approach [3,4,8]. Our case presented with a sigmoid colon obstruction complication that required surgical intervention. The cause of this case's sigmoid colon duplication remains unknown and could possibly be due to one of the theories discussed.

## Conclusions

Resecting alimentary tract duplications is a rare treatment, but it may be necessary to eliminate or avoid complications that may arise. Alimentary tract duplications are sparsely reported in the literature. Most cases represent infants less than two years of age, and adult cases are especially rare. This case report emphasizes the possible relevance and importance of awareness of an alimentary tract duplication of the sigmoid colon in an elderly patient. Robotic sigmoidectomy was chosen as the surgical modality due to patient-specific preoperative assessments. Alimentary tract duplication should be noted as a differential diagnosis for bowel obstruction in elderly patients.
